# Vaccination in the Light of the Royal British Commission

**Published:** 1896-12

**Authors:** M. R. Leverson

**Affiliations:** Fort Hamilton, Long Island


### VACCINATION IN THE LIGHT OF THE ROYAL BRITISH COMMISSION.

M. R. Leverson, M. D., Fort Hamilton, Long Island.

"Nothing is settled until it is rightly settled." Until a few years ago there was so large a consensus of medical opinion on the question of vaccination that its value as a prophylactic against small-pox was generally regarded as settled. It is true that old and experienced physicians were generally less positive upon the subject than young men fresh from college, and some men of unquestionable scientific eminence denied the alleged prophylaxis in fact, and also denied that there was any scientific basis for either the theory or practice of vaccination.

Undeniable and cumulative evidence of grave disasters following the practice added to the opposition, until at last it attained a strength such that in 1889 a Commission was appointed by the British Government, "To inquire into the effect of vaccination in reducing the prevalence of and mortality from small-pox."

The appointment of this Commission was agreed to, in the confident belief that it would put an end to the complaints and opposition of the "anti-vaccination cranks," by exposing the groundlessness of their objections.

It is proposed in this article to lay before the readers of The Homœopathic Physician the substance of the evidence on both sides; then, by way of aiding the reader to a conclusion, I will give a short account of Jenner, of his reputed discovery, and of the adoption of vaccination, and the conclusions to which the evidence seems to point.

The substance of the evidence shall be set forth, but I cannot pretend to be now without an opinion; that opinion shall be clearly stated as the result of that evidence upon a mind anxious for the truth, but entering upon the investigation *biased in favor of vaccination*, as are the minds of nearly all physicians as the result of what they are taught in their medical schools.

It is a significant fact that nearly the whole of the evidence given in support of vaccination was official evidence. Such testimony, when the official recording or delivering it has no interest in the result, deserves and always receives a large amount of credence; unhappily, where the official is interested in the result, it is of the weakest.

The marvelous genius of Bentham exactly delineated the strength and weakness of such testimony in his incomparable treatise upon evidence. (Vol. VI, Tit., Office Evidence.)

His analysis of official testimony, written at the beginning of this century and applicable to *all* such testimony, might have been a generalization from the official testimony given to the British Commission on Vaccination of 1889. Falsification of records, suppression of documents, allegation of non-existent facts, juggling with statistics, assumption of knowledge by ignorance in official position—such are the defects to which Bentham showed official evidence to be always liable, and such are the features presented by nearly all the official witnesses before the Commission.

One particular exception should be mentioned. Dr. Robert Cory gave his evidence in good faith. He was and is one of the most trusted medical officers of the local government board, the English analogue of our health boards, and is a chief instructor in vaccination. So thoroughly satisfied was he that syphilis could not be invaccinated that he made himself the subject of experiments, with the result that he invaccinated syphilis in himself!

One of the best instructed of the government officials, in the course of his testimony he makes numerous statements with regard to the nature, character, mode of operation, and results of vaccination. He makes these statements in perfect good faith as an expert, more than ordinarily competent as such by knowledge and experience to form and express an opinion, upon these subjects special within that department of medical learning to which his whole attention has been devoted.

As if to accentuate the absolute ignorance of the best instructed experts as to what they are doing in vaccination, this zealous, able, and upright gentleman—Dr. Cory—continually contradicts at one session of the Commission what he had said at a previous one; and at last, confronted with his contradictory opinions upon the subject of the relative results and dangers of the (so-called) calf lymph, or humanized lymph, he breaks down, and says (Q. 4805, p. 152b, of the second report of the Royal Commission of 1889, published in 1890):

"I am afraid that I have contradicted myself there, as in many other places."

Dr. Cory, though a prejudiced, was an honestly minded witness, but what shall be said of Sir John Simon, K. C. B., F. R. S., whose mildest terms for the opponents of vaccination have been *ignorant, idiotic, dishonest, quacks,* etc. Presenting to the Commission what he pretended was a copy of the Report of the Royal College of Physicians of England of 1807, presenting with it four of the five reports of the colleges which were embodied in that report as appendices to it, he deliberately suppressed the report of the College of Surgeons of England, which was the only one of the five reports embodied in the report of the College of Physicians which made any pretense to be based upon investigation.

The investigation, which formed the basis of the report of the College of Surgeons, thus suppressed, *was fatal to the claims of the vaccinationists!*

A pledge was given, on behalf of Sir John Simon, that he would attend the Commission and give an explanation of this suppression, but he failed to do so.

Sir John Simon had recommended Jenner's "Inquiry" to be studied by all medical men, "as a model of medical induction." At the time he made this recommendation the "Inquiry" was inaccessible to ninety-nine-hundredths of medical men; but, since 1888, it is no longer so. In his classical *History and Pathology of Small-pox,* Dr. Crookshank reproduced the "Inquiry" in full, and thus rendered it accessible to every student.

When questioned upon the contents of the work on which the whole theory of vaccination is founded, Sir John Simon professed himself unable to say whether or not Jenner considered horse-grease an essential element of true cow-pox!

Sir John Simon is equally unreliable as a statistician. Greedily swallowing the absurdities put forward as to the prevalence of small-pox in the past, he states (Q. 134, first report of the Commission, 1889, p. 6a) that in the last century almost every one had small-pox sooner or later, and that one-sixth of all who had it died, so that one-sixth of the population was continually carried off by it (Q. 137, p. 6b, *ubi sup.*).

Now, not to speak of the plague, which has not reappeared since 1680, the London bills of mortality show an annual death-rate, from fevers alone, of much more than double that of small-pox, the smallest knowledge of statistics would have shown him the impossibility of such a statement being true without a complete depopulation, not merely of the British metropolis, but of the whole of England and Wales, in a very short period!

It was admitted by all the witnesses who upheld vaccination that the mortality from fevers and zymotic diseases had largely diminished, and that while a small part of this diminution was to be ascribed to improved treatment, the decline was chiefly owing to improved sanitation; yet Sir John Simon (Q. 220–1 *ubi sup.*, p. 8a) puts the whole decline of small-pox down to vaccination!

In answer to question 237, p. 10a, Sir John Simon equivocates in a manner to excite disgust, being referred to a table of Dr. Farr, put in by himself (*Appendix*, No. 1, p. 88, *ubi sup.*), in support of a statement of that eminent statistician with regard to the decline of small-pox prior to the introduction of vaccination, and from which Sir John Simon had dissented, instead of answering with regard to the lines relating to fevers and small-pox, he runs off to the third line relating to scarlet fever only, and says: "I observe a note of interrogation in several of the columns." This very table had been referred to by him as exhibiting a decline in small-pox, and he shuffles and shuffles under the examination of Dr. Collins, reminding one forcibly of the aphorism of William Cobbett, the personification of plain common sense, when speaking of Edward Jenner himself: "Quackery has always one shuffle left."

Sir John Simon adopted a table, prepared by Dr. Ogle (Table B, p. 114, first report as above), for the purpose of showing a regular decline in the mortality from small-pox following, and *ergo*, consequent upon vaccination, in England and Wales.

He started with a septennial period, 1847–53, with a mortality per million living of 304, and then divided the following 34 years into two *unequal* periods of 18 and 16 years respectively. Thus juggled with, they produce the following table:

Mean annual deaths from small-pox *per million* living in England and Wales: 1847–53, 305; 1854–71, 223; 1872–87, 114. But now, starting with the septennial period chosen by Sir John, let us divide the remaining period also into septennials and we get the following: 1847–53, 305; 1854–60, 194 6-7; 1861–67, 181 6-7; 1868–74, 330!; 1875–81, 82 4-7; 1882–87 (five years), 53.5. (Calculated from Table A, p. 114, *ubi sup.*)

But even taking the irregular periods selected by Dr. Ogle, the fall in mortality from fevers is at least as striking in those same periods. (See Table C, p. 114, first report, *ubi sup.*)

Mean annual deaths from fever per million living in England and Wales: 1847–53, 1,139; 1854–71, 870; 1872–87, 367. This fall is unquestionably due to improved sanitary conditions, and is so admitted by Sir John Simon, vaccinationists and anti-vaccinationists; but the fall in the death-rate from small-pox is incontinently ascribed to vaccination. "Speaking broadly," says Sir John Simon, "I look to it entirely as a matter of vaccination." (Q. 221, p. 9, *ubi sup.*)

Most of the government or pro-vaccination witnesses profess to accept the opinion of Dr. Ballard, "That vaccination is not a thing to be trifled with or to be made light of; it is not to be undertaken thoughtlessly or without due consideration of the condition of the patient, his mode of life, and the circumstances of season and of place. Surgeon and patient should both carry in their minds the regulating thought that the one is engaged in communicating, the other in receiving into his system, a REAL disease—as truly a disease as small-pox or measles; a disease which, mild and gentle as its progress may be, yet, nevertheless, now and then, like every other exanthematous malady, asserts its character by an unusual exhibition of virulence."

The question must at once arise in every mind able to reason, Then why vaccinate at all? Why give a *certain* disease which "now and then asserts its character by an unusual exhibition of virulence" in the hope of charming away a disease which leaves the large majority of people unattacked, and which the "great physician," as Sydenham was rightly called, described as the mildest of diseases?

[TO BE CONTINUED.]

